# Measles case, immunization coverage and its determinant factors among 12–23 month children, in Bassona Worena Woreda, Amhara Region, Ethiopia, 2018

**DOI:** 10.1186/s13104-019-4104-8

**Published:** 2019-02-01

**Authors:** Ayele Mamo Abebe, Tamiru Mengistu, Abinet Dagnaw Mekuria

**Affiliations:** 1 Department of Nursing, Debre Birhan Health Sciences College, P.O. Box 37, Debre Berhan, Amhara Region Ethiopia; 2 School of Public Health, Debre Birhan University, Debre Berhan, Amhara Region Ethiopia

**Keywords:** Measles case, Unimmunized, Outbreak, Immunized and suspected cases

## Abstract

**Objective:**

The aims of this study were: (1) to calculate measles vaccination coverage and characterize its determinants, and (2) to qualitatively explore factors associated with reasons associated with not immunization a child.

**Result:**

In this study, the measles immunization coverage was 71.3%. The main reasons for not using the immunization services were lack of knowledge about immunization, no faith on immunization, fear of side effects and place of the service is too far. Age of mother, awareness about measles immunization, ante natal care service utilization and health facility availability were the factors that significantly associated with measles immunization. The findings of this study revealed that the coverage of measles immunization is low. Therefore health education on measles should be given for community and mothers and other additional measures should be done.

**Electronic supplementary material:**

The online version of this article (10.1186/s13104-019-4104-8) contains supplementary material, which is available to authorized users.

## Introduction

Measles is one of the leading causes for child morbidity and mortality in the world, every year around 100,000 of children lost their life due to the disease [1, 2]. In developed countries immunization coverage is a good level but in developing countries, particularly in Sub-Saharan region vaccination coverage is very low [3–6]. Demographic health survey data shows in low and middle income countries that only 50% of the population vaccinated for measles and measles outbreak evidenced in these countries during 2016; 4395, 1500, and 162 measles cases occur in Ethiopia, South Sudan and Kenya, respectively [7–9]. Millions of individuals still don’t protected by the vaccine regardless of many effort done by gov’t [10, 11].

In many developing countries because of poor measles immunization coverage outbreaks of the disease are common and it puts enduring infirmities such as brain impairment, blindness, and deafness [10–12]. It is also responsible for 5% of the overall mortality from diseases which can be potentially prevented by vaccine [12].

A baseline survey in selected zones of Ethiopia shows that valid vaccination coverage for measles was 50% and timely valid dose provided before the age of 12 months for measles was found to be 39% [4]. There are different factors associated with immunization coverage in the Africa as well as in Ethiopia among those factors access to the immunization services, caregivers awareness, time of vaccination not known and long distance to reach institutions, maternal level of education, exposure to media, and antenatal care service utilization, geographic variations, and socio-cultural beliefs coupled with the timing of the measles vaccine as well as the fragile health service infrastructure [13–18]. Studies also evidenced inadequate immunization for measles will greatly affect the health of children [15, 19].

The Bassona Worena Woreda vaccination coverage not achieve the WHO targets of 80% immunization coverage by 2015. Therefore, this study was used to determine measles case, immunization coverage and its determinant factors between 12 and 23 months child in Bassona Worena Woreda, North Shoa Zone, Ethiopia.

## Main text

### Study area and period

The study was conducted in the Bassona Worena which is one of 24 Woredas in North Shoa zone, Amhara region. Bassona Worena Woreda is located in Northern part of Addis Ababa at 130 km distance. The main ethnic group is Amhara in 2017, an estimated population of children’s under 2 years old was 7520. The study was conducted from April 1–30, 2018.

#### Study design

The community based mixed cross sectional study design was conducted.

#### Study population

All 12–23 months old children living in the selected clusters in the Bassona Worena Woreda.

#### Sample size determination

The sample size was determined by using world health organization expanded program of immunization coverage cluster survey. The percentage of measles coverage(p) = 77% [15]. The WHO cluster survey sample size calculation method formula, n = z^2^ * p * q * DEFF/d^2^ utilized with the assumption of:

Z: the Z-score at 95% confidence interval which is 1.96.

DEFF: the design effect of 2.

d: the tolerable margin of error which is 5%.

Then: n = 1.96^2^ * 0.77 * (1 − 0.77) × 2/(0.05)^2^

n = 544.

By adding 10% (54) nonresponse rate the total sample become 598.

#### Sampling technique

**Fig. 1:**
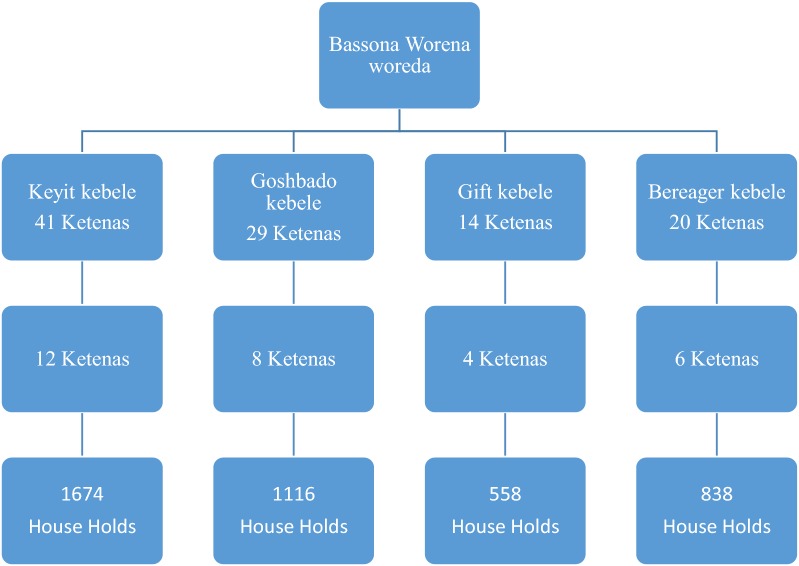
Schematic representation of the sampling procedure

The sample size was calculated based on the WHO 30 cluster based survey method. There were 4 clusters (Keyit, Goshbado, Gift and Bereager) in Bassona Worena woreda. In the woreda there were a total 31 kebeles that comprise 104 ketena. A total of 30 ketenas were selected by using simple random sampling technique from each cluster kebeles. Households in each ketenas were selected, through random selection (Fig. 1).

#### Operational definition

##### Measles immunized

A 12‒23 months old child who received one dose of measles [15].

##### Measles unimmunized

A 12‒23 Months old child who did not receive any dose measles vaccines [15].

A confirmed measles outbreak, is defined as the occurrence of three or more confirmed measles cases [15].

### Data collection tools and procedure

#### Quantitative method

An interview based structured questionnaire was developed after revising previous studies [5, 15]. Six data collector and two supervisors were trained about the data collection way.

### Qualitative method

The questionnaire was first prepared in English language then it translated to Amharic language and guide translated back to English to check the effectiveness questionnaire. The different groups were selected purposively for focus group discussion. Five focus group discussions, two with health professionals working in EPI unit and three with women with children conducted and then information saturation obtained.

#### Data quality assurance

The English language questionnaire was translated into Amharic language to check the consistency of the tool. The data collection tool was pre tested on 42 households in one randomly selected kebele which is not included in the study. Training was given for data collectors and supervisors. Finally, the data collectors checked the completeness of the questionnaire at the end each day of data collection (Additional file 1).

#### Data processing and analysis

##### For the quantitative part

Finally, data checked, coded and entered into Epi data software. SPSS version 20 was used to analyze the collected data. The data described by computing frequency, tables, graph and statistical summaries. Variables showed statistical significance during bivariate analysis at P-value < 0.20 were entered to multivariable logistic regression. Adjusted odds ratios (AOR) with 95% CI, were estimated to assess the strength of associations and statistical significance was declared at P-value < 0.05.

##### For the qualitative part

The qualitative data was analyzed by employing thematic analysis. The main ideas of the discussion extracted from audio record and written data and it grouped under each items of discussion guide. After the main ideas become organized, thematic areas developed, then ideas categorized under each thematic areas and it coded. The result of the qualitative part presented and discussed by triangulating with the quantitative part.

### Result

#### Maternal or caregivers socio-demographic characteristics

A total of 575 women interviewed; making response rate of 96.2%. About 80% of the participants were rural resident. About, 259 (45.0%) were in the age group of 20–29 years, the median age was 30 years with IQR of 9 years. Majority 506 (88.0%) of the respondents were Orthodox and 79.3% of them were Amhara in ethnicity. Around 75.0% of respondents are married and most of them (57.3%) of them had monthly income of above 1000 Birr and (37.0%) of respondents were illiterate (Table 1).

**Table 1:** Socio-demographic characteristics of respondents, Bassona Worena Woreda, North Shoa zone, Ethiopia 2017 (n = 575)

Characteristics	Frequency	Percent (%)
Age in years		
Less than 20	23	4.0
20–30	259	45.0
30–39	211	35.7
Above 40	88	15.3
Religion		
Orthodox	506	88.0
Protestant	63	11.0
Muslim	6	1.0
Ethnic group		
Amhara	456	79.3
Oromo	117	20.3
Tigrey	2	0.3
Marital status		
Single	75	13.0
Married	433	75.3
Windowed	52	9.0
Divorced	16	2.7
Monthly income (birr)		
Less than 1000	243	42.3
Above 1000	332	57.7
Educational status		
Illiterate	213	37.0
Read and write	117	20.3
Grade 1–8	144	25.0
Preparatory and above	101	17.6

#### Service utilization related factors

From the total, 312 (54.3%) did not attend ANC during their last child birth. Among respondents who attend ANC only 37.3% of them attend once. The majority 510 (88.7%) of respondents did not know any child with abscess after immunization (Table 2).

**Table 2:** Service utilization related factors of respondents, Bassona Worena Woreda, North Shoa zone, Ethiopia 2017 (n = 575)

Service utilization factors	Frequency	Percent (%)
ANC		
Yes	263	45.7
No	312	54.3
Number of ANC		
1	214	37.3
2	46	8.0
4	3	0.3
Know child with abscess		
Yes	65	11.3
No	510	88.7
Whose child		
Own child	23	4.0
Neighbor child	33	5.7
Friend child	10	1.7
Abscess location		
Arm	56	9.7
Thigh	10	1.7

#### Institutional factors

According to this study, 458 (79.7%) of respondents explained that there was a health facility that provide measles immunization and health post were the frequently 315 (54.7%) reported health facility. Two hundred thirty-three (41.0%) of respondents said 15–30 min required to reach the health facility. Majority of them (71.0%) said the quality of the services provided was good (Additional file 2: Table S1).

FGD participants indicated the availability of adequate number of health institutions providing measles immunizations services in the woreda. During the discussion, all FGD participants agreed as the quality of measles immunization service provided was good.

#### Child related factors

From the total participants, 414 (72.0%) of the child are in the age group of 12–23 months and 51.3% of them were female. Most of them (61.0%) had less than 3 siblings (Additional file 3: Table S2).

#### Knowledge of mothers or caregivers

In this study, 439 (76.3%) of mothers ever heard about measles immunization. The main source of information was health professionals (47.7%). Two hundred forty-seven (43.0%) of the respondents said that the objective of immunization was to prevent measles. Two hundred twenty-four (39.0%) of respondents measles immunization given at 9 months (Additional file 4: Table S3).

### Measles case

It was found that none of the respondents reported the measles symptoms seen in the child in the past 2 months.

### Measles immunization

According to this study, 71.3% of child receives measles immunization and most (51.0%) of them receive in health posts, 19.3% at health centers and 1.0% at hospital (Additional file 5: Figure S1). Approximately thirty percent of participants explained why not immunized their children that I do not know about vaccine was the most common reason (Additional file 6: Figure S2).

In FDG with midwifery, one midwifery nurse working in ANC department explained that:

*“All pregnant women who come to our health centers will be given health education about their child immunization and other women who came from rural kebeles will be linked with health extension workers.”*

#### One rural women FGD participant said that

*“I think the reason for not immunizing their child is that some women are far from health facility this is very challenging for those women to come to health facilities.”*

#### Factors associated with measles immunization

In multi-variate logistic regression analysis only age of mothers, use of ANC service awareness and health facility availability were the factors that significantly associated with measles immunization. Age group of 40 and above (AOR = 1.9; 95% CI 1.12, 5.83), awareness about measles immunization (AOR = 2.8; 95% CI 1.67, 9.34), ANC use (AOR = 3.67; 95% CI 1.96, 6.78) and health facility availability (AOR = 1.49; 95% CI 1.06, 8.12) were the independent predictors of measles immunization (Additional file 7: Table S4).

### Discussion

This study was conducted to determine measles immunization coverage and its associated factors among 12–23 months children. The measles immunization coverage in this study was 71.3% which is comparatively smaller than studies done in Lay Armachiho District North Gondar Zone (77%) and Malawi (95.0%) [15, 20]. This discrepancy might be due to socio-cultural variation among those countries.

In this study, mothers in who were 40 years and above were 2 times more likely than other age groups. This finding was consistent with a Bangladesh study [21].

The main reasons for not immunizing child in this study were lack of awareness about immunization and no faith in immunization. During the FGD with HCW the existence of inadequate scientific knowledge about measles in some women was mentioned. This finding was similar to study conducted in Somalia. But study conducted in Congo found lack knowledge was the main reason reported by mothers who did not immunize their child for measles [22].

Mothers who were aware of measles immunization importance were 2.8 times more likely to immunize their childhan children of mothers who had poor knowledge on measles vaccine. This finding was similar with studies conducted in different areas [22–25]. This result supported by the findings of the qualitative parts of the study during the FGD with HCW the existence of inadequate scientific knowledge about measles in some women was mentioned.

Based on this study, mothers who had utilized ANC during the pregnancy were 3.67 times more likely to immunize their children than mothers who have not utilized. This result is supported by the qualitative part of the study. This finding was aligned with studies done in Amibara district and North Indian founds [26, 27].

According to this study, mothers who said health facility are available were 1.49 times more likely to be measles vaccinated than those who said unavailable of health facility. This finding was consistented with study in Amibara District [26]. This finding was supported by qualitative part of this study.

Similar to current study findings in an affluent community in the United States of America identified fear of side effects as an important factor for low coverage of immunization when they conducted [28, 29].

### Conclusions

In this study, the coverage of measles immunization is low. Age of mother, awareness about measles immunization, ANC service use and health facility availability were the factors that significantly associated with measles immunization. The main reasons for not using the immunization services were lack of knowledge about immunization, no faith on immunization, fear of side effects and far place of the service. Therefore health education on measles should be given for community and mothers and other additional measures should be done.

### Strength of the study

The study design was mixed cross sectional study method. It was useful to dig out the most important factors that affect the measles immunization coverage.

## Limitation of the study

The use of cross-sectional study design couldn’t formulate a temporal relationships between cause and effect.

## Additional files

**Additional file 1.** English version questionnaire for measles.

**Additional file 2: Table S1.** Institutional related factorof respondents, Bassona worena woreda, North Shoa zone, Ethiopia 2017 (n = 575).

**Additional file 3: Table S2.** Child related factor of respondents, Bassona worena woreda, North Shoa zone, Ethiopia 2017 (n = 575).

**Additional file 4: Table S3.** The knowledge of respondents, Bassona worena woreda, Ethiopia 2017 (n = 575).

**Additional file 5: Figure S1.** Measles immunization among 12 to 23 months old child, Bassona Worena woreda, Ethiopia, 2017.

**Additional file 6: Figure S2.** Reasons for not immunizing child among respondents, Bassona Worena woreda, Ethiopia, 2017.

**Additional file 7: Table S4.** Binary logistic regression analysis of factors associated with measles immunization, Bassona worena woreda, Ethiopia 2017 (n = 575).

## Abbreviations

ANC: antenatal care
BCG: Bacillus Chalmette–Guerin vaccine
CDC: Central Disease Control
CSA: Central Statically Agency
CMYP: comprehensive multi year plan
EDHS: Ethiopian Demographic and Health Survey
EFY: Ethiopia Fascia Year
EHNRI: Ethiopian Health and Nutrition Research Institute
EPHI: Ethiopia Public Health Institution
EPI: expanded program for immunization
ERIA: enhanced routine immunization activities
FMoH: Federal Ministry of Health
HSDP: Health Sector Development Program
HMIS: Health Management Information System
MCV: measles-containing vaccine
NGO: non-governmental organization
RED: reaching every district
RIIP: routine immunization improvement plan
SIAs: supplemental immunization activities
SNNP: Southern Nations and Nationality of Peoples
UNICEF: United Nations Children’s Fund
VPD: vaccines preventable disease
WHO: World Health Organization
