# Supplementary material for: Measles case, immunization coverage and its determinant factors among 12–23 month children, in Bassona Worena Woreda, Amhara Region, Ethiopia, 2018
Source: BMC Res Notes. 2019 Feb 1;12:71. doi: 10.1186/s13104-019-4104-8 (PMC6359826; doi:10.1186/s13104-019-4104-8)
Supplement: Supplementary file 1 — Additional file 1. English version questionnaire for measles. [file 13104_2019_4104_MOESM1_ESM.docx]

## English version questionnaire for measles

**Part one: Socio-demographic characteristics of mothers or care givers**

| No | Questions | Options/ responses | Skip to |
| --- | --- | --- | --- |
| 101 | How old are you? | _______________ years |  |
| 102 | What is your religion? | 1. Orthodox 2. Muslim 3. Protestant 4. Other specify ___________ |  |
| 103 | What is your ethnic origin? | 1. Amahara 2. Oromo 3. Tigrey 4. Other (specify) ____________ |  |
| 104 | What is your Marital status? | 1. Married 2. Single/never married 3. Divorced 4. Widowed 5. Separated |  |
| 105 | What is the average monthly income of the household? | __________________ Birr |  |
| 106 | What is your educational status? | 1. Illiterate 2. Read and write 3. Grade 1 to 8 4. Grade 9 to 12 5. College/ university |  |

**Knowledge of mothers or care givers on measles immunization**

| No | Questions | Options or responses | Skip to |
| --- | --- | --- | --- |
| 107 | Do you heard about measles immunization? | 1. Yes 2. No | 109 |
| 108 | If yes, where do you heard about measles immunization? | 1. Radio/ television 2. Friends/ peer 3. School 4. Health professionals 5. Others (specify) ____________________ |  |
| 109 | Do you mention the objective of vaccinating child for measles? | 1. To prevent diseases 2. To prevent measles 3. I do not know 4. Other (specify) ____________________ |  |
| 110 | Do you tell me the age at which the child will take measles immunization? | 1. Just after birth 2. One month after birth 3. At nine month 4. After one year 5. I do not know 6. Others (specify) ________________ |  |
| 111 | How many times should a child receive measles immunization? | 1. One 2. Three 3. Five 4. I do not know |  |
| 112 | Do you think vaccination for measles will make your child sick | 1. Yes 2. No 3. I do not know |  |
| 113 | Do you think a sick child should receive measles vaccination? | 1. Yes 2. No 3. I do not know |  |

**Part two: Service utilization related factors**

| No | Questions | Options or responses | Skip to |
| --- | --- | --- | --- |
| 201 | Did you attend antenatal care during your last pregnancy? | 1. Yes 2. No | 203 |
| 202 | If yes, how many times did you attend antenatal care? | 1. One 2. Two 3. Three 4. More than three |  |
| 203 | Do you know of any child who had an abscess after a vaccination? | 1. Yes 2. No | 206 |
| 204 | Who was the child? | 1. Own child 2. Neighbor child 3. Friend's child 4. Family member's child 5. Other (specify)__________ |  |
| 205 | Where was the abscess located? | 1. Arm 2. Thigh 3. Other (specify) _____________ |  |
| 206 | If your child was due for a vaccination and was showing symptoms of a fever, would you take them to be vaccinated? | 1. Yes 2. No |  |
| 207 | If your child was due for a vaccination and if they had a cough? | 1. Yes 2. No |  |
| 208 | If your child was due for a vaccination and if they had a rash? | 1. Yes 2. No |  |

**Part three: Child related factors**

| No | Questions | Options or responses | Skip to |
| --- | --- | --- | --- |
| 301 | What is the child birth date in day? | ______________ Day |  |
| 302 | What is the child birth date in month? | ______________ Month |  |
| 303 | What is the child birth date in year? | _____________ Year |  |
| 304 | What is the sex of the child? | 1. Male 2. Female |  |
| 304 | What is your family size? | _____________________ |  |
| 305 | What is the number of children siblings? | 1. One 2. Two 3. Three 4. More than three |  |
| 306 | At what age you give the first birth? | ___________________ Year |  |
| 307 | How many months did you stay before the next pregnancy? | __________________ Months |  |
| 308 | Where did you deliver your last child? | 1. Home 2. Health facility 3. Other (specify) __________________ |  |

**Part four: Service utilizations related to factors**

| No | Questions | Options or responses | Skip to |
| --- | --- | --- | --- |
| 401 | Is there any health facility which provides measles immunization service near to you? | 1. Yes 2. No 3. I do not know | 501 |
| 402 | If yes to the above question, which health facility is near to you? | 1. Health center 2. Hospital 3. Health post 4. Private health institutions |  |
| 403 | How many minutes does it take to reach to the health facility? | 1. Less than 15 minutes 2. 15 to 30 minutes 3. 30 to 60 minutes 4. More than 60 minutes 5. I do not know |  |
| 404 | Do you think about the immunization services quality provided is good? | 1. Yes 2. No 3. I do not know | 501 |
| 405 | If No to the above questions, what is your reason? |  |  |

**Part five: Child measles immunization**

| No | Questions | Options or responses | Skip to |
| --- | --- | --- | --- |
| 501 | Has the child ever received vaccine given by injection on the left upper arm? | 1. Yes 2. No | 505 |
| 502 | How many times was measles vaccine given? | _____________________ |  |
| 503 | At what age the child first receive measles vaccine? | ________________ Months |  |
| 504 | Where did your child receive the measles vaccine? | 1. Health post 2. Health center 3. Hospital 4. Private health institutions 5. Other (specify) _________________ |  |
| 505 | Why hasn't the child had measles vaccines? | 1. Place of immunization too far 2. Time of immunization inconvenient 3. Mother too busy 4. Family problem, including illness of mother 5. No faith in immunization 6. Fear of side reactions 7. Place and/or time of immunization unknown 8. Others (specify) __________________ |  |

**Part six: Measles case assessment questions**

| No | Questions | Options or responses | Skip to |
| --- | --- | --- | --- |
| 601 | Did the child have any of the following symptoms with in past two month? | | |
|  | Fever: | 1. Yes 2. No |  |
|  | Runny nose: | 1. yes 2. No |  |
|  | Red eyes: | 1. Yes 2. No |  |
|  | Cough : | 1. yes 2. No |  |
|  | Tiny white spots or sores inside the mouth | 1. yes 2. No |  |
|  | Rash | 1. Yes 2. No |  |

**This is the end the interview thank you for your time!**
