# Supplementary material for: Measles case, immunization coverage and its determinant factors among 12–23 month children, in Bassona Worena Woreda, Amhara Region, Ethiopia, 2018
Source: BMC Res Notes. 2019 Feb 1;12:71. doi: 10.1186/s13104-019-4104-8 (PMC6359826; doi:10.1186/s13104-019-4104-8)
Supplement: Supplementary file 2 — Additional file 2: Table S1. Institutional related factorof respondents, Bassona worena woreda, North Shoa zone, Ethiopia 2017 (n = 575). [file 13104_2019_4104_MOESM2_ESM.docx]

Table S1: Institutional related factor of respondents, Bassona worena woreda, North Shoa zone, Ethiopia 2017 (n= 575)

| **Institutional related factors** | **Frequency** | **Percent (%)** |
| --- | --- | --- |
| Is there a facility for immunization |  |  |
| Yes | 458 | 79.7 |
| No | 12 | 2.0 |
| I do not know | 105 | 18.3 |
| Which health facility |  |  |
| Health center | 140 | 24.3 |
| Health post | 315 | 54.7 |
| hospital | 6 | 1.0 |
| Minutes to health facility |  |  |
| Less than 15 min | 59 | 10.3 |
| 15 to 30 min | 236 | 41.0 |
| Above 30 min | 165 | 28.7 |
| I do not know | 2 | 0.3 |
| Quality of the services good |  |  |
| Yes | 408 | 71.0 |
| No | 4 | 0.7 |
| I do not know | 53 | 9.3 |
