# Supplementary material for: Measles case, immunization coverage and its determinant factors among 12–23 month children, in Bassona Worena Woreda, Amhara Region, Ethiopia, 2018
Source: BMC Res Notes. 2019 Feb 1;12:71. doi: 10.1186/s13104-019-4104-8 (PMC6359826; doi:10.1186/s13104-019-4104-8)
Supplement: Supplementary file 3 — Additional file 3: Table S2. Child related factor of respondents, Bassona worena woreda, North Shoa zone, Ethiopia 2017 (n = 575). [file 13104_2019_4104_MOESM3_ESM.docx]

Table S2: Child related factor of respondents, Bassona worena woreda, North Shoa zone, Ethiopia 2017 (n= 575)

| **Child related factors** | **Frequency** | **Percent (%)** |
| --- | --- | --- |
| Months of child |  |  |
| Less than 12 months | 161 | 28.0 |
| 12 to 23 months | 414 | 72.0 |
| Sex of the child |  |  |
| Male | 280 | 48.7 |
| Female | 295 | 51.3 |
| Family size |  |  |
| less than 5 | 142 | 24.7 |
| 5 and above | 433 | 75.3 |
| Number of siblings |  |  |
| No sibling | 58 | 10.0 |
| Less than 3 | 351 | 61.0 |
| 3 and above | 167 | 29.0 |
