# Supplementary material for: Measles case, immunization coverage and its determinant factors among 12–23 month children, in Bassona Worena Woreda, Amhara Region, Ethiopia, 2018
Source: BMC Res Notes. 2019 Feb 1;12:71. doi: 10.1186/s13104-019-4104-8 (PMC6359826; doi:10.1186/s13104-019-4104-8)
Supplement: Supplementary file 4 — Additional file 4: Table S3. The knowledge of respondents, Bassona worena woreda, Ethiopia 2017 (n = 575). [file 13104_2019_4104_MOESM4_ESM.docx]

Table S3: the knowledge of respondents, Bassona worena woreda, Ethiopia 2017 (n= 575)

| **Knowledge** | **Frequency** | **Percent (%)** |
| --- | --- | --- |
| Ever heard |  |  |
| Yes | 439 | 76.3 |
| No | 136 | 23.7 |
| Source of information |  |  |
| Radio | 13 | 2.3 |
| Friend | 151 | 26.3 |
| Health professionals | 274 | 47.7 |
| Objective of immunization |  |  |
| To prevent disease | 69 | 12.0 |
| To prevent measles | 247 | 43.0 |
| I do not know | 122 | 21.3 |
| Age of immunization |  |  |
| At birth | 56 | 9.7 |
| At one month | 67 | 11.7 |
| At nine month | 224 | 39.0 |
| I do not know | 92 | 16.0 |
| Number of immunization |  |  |
| One | 320 | 55.7 |
| Three | 29 | 5.0 |
| Five | 2 | 0.3 |
| I do not know | 88 | 15.3 |
