# Supplementary material for: Measles case, immunization coverage and its determinant factors among 12–23 month children, in Bassona Worena Woreda, Amhara Region, Ethiopia, 2018
Source: BMC Res Notes. 2019 Feb 1;12:71. doi: 10.1186/s13104-019-4104-8 (PMC6359826; doi:10.1186/s13104-019-4104-8)
Supplement: Supplementary file 7 — Additional file 7: Table S4. Binary logistic regression analysis of factors associated with measles immunization, Bassona worena woreda, Ethiopia 2017 (n = 575). [file 13104_2019_4104_MOESM7_ESM.docx]

Table S4: Binary logistic regression analysis of factors associated with measles immunization, Bassona worena woreda, Ethiopia 2017 (n= 575)

| **Variables** | **Immunized n** | **COR (95% CI)** | **AOR (95% CI)** |
| --- | --- | --- | --- |
| Age in years |  |  |  |
| Less than 20 | 14 | 1 | 1 |
| 20 to 29 | 162 | 0.57 (0.352, 0.929) | 0.03 (0.02, 0.463) |
| 30 to 39 | 166 | 0.649 (0.402, 1.049) | 0.06 (0.01, 0.313) |
| 40 and above | 68 | **2.2 (1.01, 5.62)*** | **1.9 (1.12,5.83) *** |
| Religion |  |  |  |
| Orthodox | 396 | 1 |  |
| Protestant | 51 | 2.571 (0.500, 13.229) |  |
| Muslim | 4 | 1.087 (0.269, 4.393) |  |
| Ethnic Group |  |  |  |
| Amhara | 310 | 1 |  |
| Oromo | 98 | 1.963 (0.403, 7.797) |  |
| Tigrey | 2 | 2.87 (0.500, 13.229) |  |
| Marital Status |  |  |  |
| Single | 40 | 1 |  |
| Married | 329 | **3.648 (2.250, 5.914) *** |  |
| Windowed | 32 | 1.749 (1.176, 2.603) |  |
| Divorced | 9 | 1.087 (0.269, 4.393) |  |
| Monthly income |  |  |  |
| < 1000 birr | 114 | 1 |  |
| >= 1000 birr | 296 | **1.980 (1.492, 2.954) *** |  |
| Educational status |  |  |  |
| Illiterate | 108 | 1 |  |
| Read and write | 102 | 1.572 (0.630, 3.921) |  |
| Grade 1 to 8 | 100 | 0.447 (0.163, 1.227) |  |
| Above second | 91 | **1.394 (0.153, 3.017) *** |  |
| Aware measles vaccine |  |  |  |
| Yes | 349 | 1 | 1 |
| No | 61 | **3.11 (1.76, 7.86)*** | **2.8 (1.67, 9.34) *** |
| ANC use |  |  |  |
| No | 176 | 1 | 1 |
| Yes | 234 | **5.282 (2.24, 12.8)*** | **3.67 (1.96, 6.78) *** |
| Facility available |  |  |  |
| No | 374 | 1 | 1 |
| Yes | 36 | **2.499 (1.796, 3.476)*** | **1.49 (1.06, 8.12) *** |

*significantly associated
