# Supplementary material for: Measles case, immunization coverage and its determinant factors among 12–23 month children, in Bassona Worena Woreda, Amhara Region, Ethiopia, 2018
Source: BMC Res Notes. 2019 Feb 1;12:71. doi: 10.1186/s13104-019-4104-8 (PMC6359826; doi:10.1186/s13104-019-4104-8)
Supplement: Supplementary file 7 [file 13104_2019_4104_MOESM6_ESM.docx]

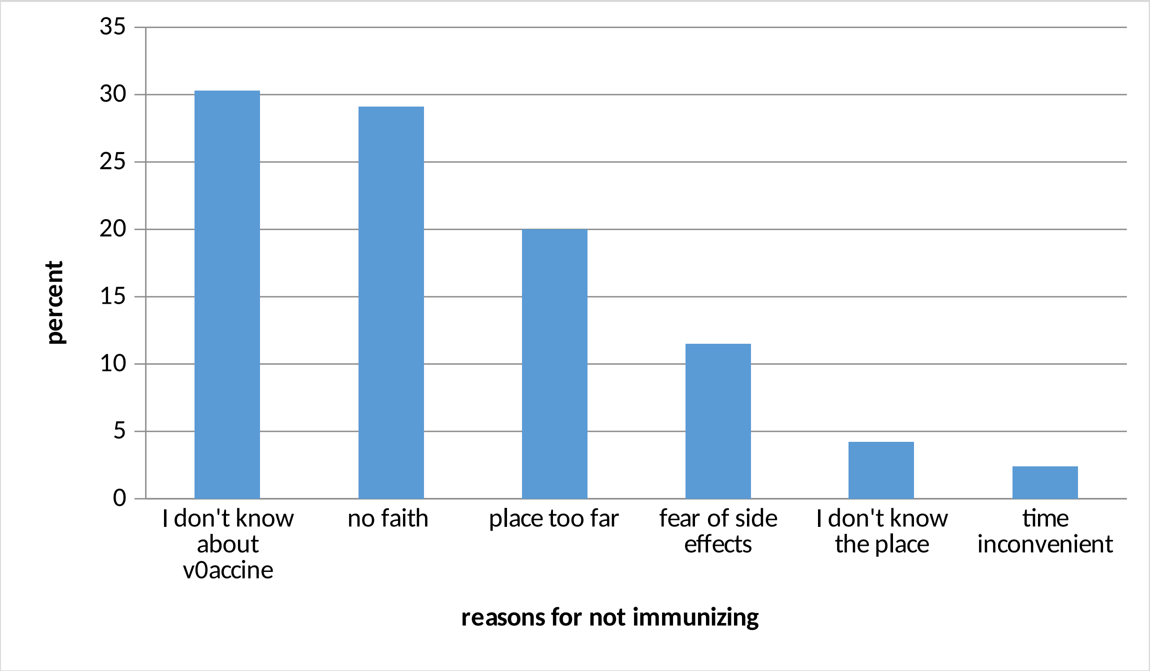

35
30
25
20
15
10
5
0
percent
I don't know about v0accine
no faith
place too far
fear of side effects
I don't know the place
time inconvenient
reasons for not immunizing
